# EFR3A, an Intriguing Gene, and Protein with a Scaffolding Function

**DOI:** 10.3390/cells14060445

**Published:** 2025-03-17

**Authors:** Magdalena Trybus, Anita Hryniewicz-Jankowska, Aleksander Czogalla, Aleksander F. Sikorski

**Affiliations:** 1 Research and Development Centre, Regional Specialist Hospital, ul. Kamieńskiego 73a, 51-124 Wrocław, Poland; magdalenatrybus89@gmail.com; 2 Department of Cytobiochemistry, Faculty of Biotechnology, University of Wrocław, ul. Joliot-Curie 14a, 50-363 Wrocław, Poland; anita.hryniewicz-jankowska@uwr.edu.pl

**Keywords:** EFR3, PI4KA, phosphatidyl inositol derivatives, PKC, membrane rafts

## Abstract

The EFR3 (Eighty-Five Requiring 3) protein and its homologs are rather poorly understood eukaryotic plasma membrane peripheral proteins. They belong to the armadillo-like family of superhelical proteins. In higher vertebrates two paralog genes, A and B were found, each expressing at least 2–3 protein isoforms. EFR3s are involved in several physiological functions, mostly including phosphatidyl inositide phosphates, e.g., phototransduction (insects), GPCRs, and insulin receptors regulated processes (mammals). Mutations in the *EFR3A* were linked to several types of human disorders, i.e., neurological, cardiovascular, and several tumors. Structural data on the atomic level indicate the extended superhelical rod-like structure of the first two-thirds of the molecule with a typical armadillo repeat motif (ARM) in the N-terminal part and a triple helical motif in its C-terminal part. EFR3s’ best-known molecular function is anchoring the giant phosphatidylinositol 4-kinase A complex to the plasma membrane crucial for cell signaling, also linked directly to the *KRAS* mutant oncogenic function. Another function connected to the newly uncovered interaction of EFR3A with flotillin-2 may be the participation of the former in the organization and regulation of the membrane raft domain. This review presents EFR3A as an intriguing subject of future studies.

## 1. Introduction

The EFR3A (Eighty-Five Requiring, homolog A) protein and its paralogs and orthologs are rather poorly understood eukaryotic plasma membrane (PM) peripheral proteins. In eukaryotic organisms, the genes encoding yeast EFR3 homologs belong to a group of genes called the armadillo-like superfamily ARMH, also called ARM-like, encoding highly helical and superhelical proteins. This superfamily is characterized by a domain consisting of a multi-helical motif composed of two curved layers of α-helices arranged in a right-handed superhelix, in which the repeats forming this structure are arranged around a common axis. (see below, Section 4).

The yeast *EFR3* (*YMR212C*) gene ortholog was originally discovered and named as one of seven Pho 85 (nonessential CDK) Requiring (*efr*) loci, *efr1–3* and *5–8* [1]. It appeared orthologous to *D. melanogaster*, the rolling blackout (rbo, stmA-PF; stambhA; cmp 44E) involved in phototransduction and synaptic vesicular traffic [2,3]. Moreover, *EFR3* ortholog genes can be found in lower metazoan organisms such as *Cnidaria*, e.g., coral *Stylophora pistillata*, and *Nematoda*, e.g., *Caenorhabditis elegans* [4]. In *Chordata,* including *Urochordata* (tunicates), *Cephalochordata* (Lancelets), and *Cyclostomata* (Lampreys), orthologs resembling homolog B in all remaining vertebrates can be found [5].

**Figure 1:**
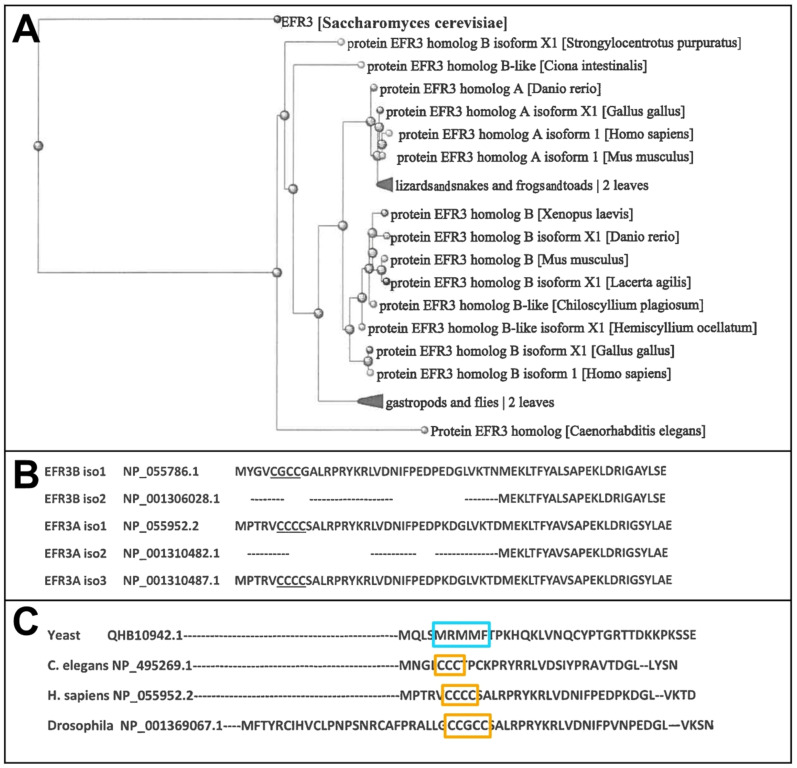
Evolution of EFR3 proteins. (**A**). Simplified phylogenic tree of EFR3 prepared with NIH BLASTp software (https://blast.ncbi.nlm.nih.gov/Blast.cgi?PAGE=Proteins&PROGRAM=blastp&BLAST_PROGRAMS, accessed on 15 December 2024). The more detailed phylogenic tree can be found under (Ensemble GeneTree ENSGT00390000002143) [6]. (**B**). Sequence comparison of EFR3A isoforms. Isoforms 2 of both EFR3A and EFR3B lack an N-terminal sequence containing a C-cluster (undelined). (**C**). Three methionine residues in yeast EFR3 (blue frame) may have preceded N-terminal cysteine cluster (3–4 residues) in metazoan orthologs (orange frames) starting from *Nematoda*. Analyses were performed by using Clustal Omega web software [7].

In all vertebrates (excluding *Cyclostomata*), two homolog genes and proteins, A and B, were found, each expressing at least 2–3 experimentally confirmed protein isoforms as well as several predicted ones (Figure 1A) that may mean that the first two paralogs might have appeared in cartilagous fish around 400 mya. In humans, two homolog genes located in chromosomes 8 and 2 encode proteins EFR3A and EFR3B, respectively. Overall homology between these two gene products is rather moderate and reaches, for example, for isoforms 1 of each protein, 64% identity and 78% overall similarity. As can be seen, isoforms 2 of either homolog lack an N-terminal sequence containing 3–4 cysteine residues, which are the target of palmitoylation (see Figure 1B and the text below). It is interesting that in yeast EFR3, a small hydrophobic three methionine and one phenylalanine residue cluster (5-MRMMF-9) can be observed (see Figure 1C). Constituting a permanent hydrophobic patch, it might participate in the interaction with the plasma membrane.

Moreover, Noack et al., while looking for possible EFR3 homologs in *Arabidopsis* via sequence alignments found four potential proteins that they called the EFR of plants (EFOP) 1–4 which belong to the class of ARMH superfamily. They found several structural and functional similarities between fungal and animal EFR3 and plant proteins [8].

The *EFR3A* gene in *Homo sapiens* is located on chromosome 8 (8q24.22) and spans 109,550 nt. The gene comprises up to 40 exons with 11 transcription variants, including 8 alternatively spliced [9]. According to this source, the *EFR3A* gene was found to be expressed to rather high levels, i.e., exceeding 4.3 times the average gene expression in normal and pathological human tissues. In turn, the gene encoding EFR3B is located on the short arm of chromosome 2, spans 117,036 nt, and contains up to 33 exons, the product of which is eight different mRNA molecules, including four representing alternatively spliced transcripts. Two isoforms of 817 and 787 amino acid residues long (isoform 1 and 2, respectively) have been listed in humans [10]. This homolog is expressed at the average level in various organs, tissues, and cells of primates and humans [9].

## 2. Physiological Roles of EFR3A

### 2.1. Drosophila Melanogaster Phototransduction

Phospholipase C (PLC)-dependent opening of transient receptor potential (TRP) and TRP-like (TRPL) channels mediate phototransduction in flies [11,12]. PLC*β* cleaves PI(4,5)P_2_ into inositol 1,4,5-trisphosphate (IP_3_) and diacylglycerol (DAG), which results in the activation of the channels mentioned above and triggers an electrical signal in response to light stimulus [13]. As the PLC activity is very high, a feedback inhibition mechanism involving Ca^2+^ and PKC maintaining the levels of PI(4)P and PI(4,5)P_2_ is suggested [14].

Reducing phosphatidylinositol 4-kinase PI4KIIIα (PI4KA) levels by knockdown or using *PI4KA* mutants results in decreased levels of PM PI(4)P and PI(4,5)P_2_ and altered light responses of *Drosophila* photoreceptors. Depletion of the *Drosophila* Efr3 (rbo) and tetratricopeptide repeat domain 7 (TTC7), which attach PI4KAα to the PM, also results in reduced PM PI(4)P and PI(4,5)P_2_ levels, and an impaired light response [15,16].

Phosphoinositides are implicated in the vesicular traffic. Data on *Drosophila* rbo mutants may confirm this hypothesis, although they indicate some differences between neuronal and non-neuronal vesicular traffic [17].

### 2.2. EFR3 Proteins Affect GPCR Responsiveness by Regulating Receptor Phosphorylation

A and B paralogs of EFR3 were found to be involved in the GPCR receptor signaling cascades [18]. The AT1R (angiotensin II receptor type 1) belongs to seven transmembrane helical G-protein-coupled receptors (GPCRs). Activation of the AT1R regulates various physiological events in different tissues, namely, vasoconstriction, aldosterone release, renal sodium reabsorption, facilitation of adrenergic transmission, vascular smooth muscle cells (VSMC) hypertrophy, and cardiac myocyte hyperplasia (for a review, see e.g., [19,20,21]). One of the several and best-known signaling pathways of the AT1R is the activation of phospholipase C via heterotrimeric Gq/11 protein (human G_α11_ encoded by the *GNA11* gene) leading to the generation of two second messengers: DAG and IP_3_. The first one activates the calcium-dependent protein kinase C (PKC), which phosphorylates several proteins, among them myosin light chain kinase or PP2Ac (a protein phosphatase), which dephosphorylates AKT and inactivates it leading to the death-receptor-independent apoptosis [22]. The second product of PI(4,5)P_2_ hydrolysis binds to IP_3_ receptors in the endoplasmic reticulum; ligand-gated Ca^2+^ channels are responsible for this ion release from this organelle functioning as intracellular calcium stores [23].

EFR3 proteins are thought to localize phosphatidylinositol 4-kinase (PI4KA) to the PM (see below). Bojjireddy et al. [18], while silencing *EFR3A* and/or *EFR3B* gene expression, found that after AngII stimulation in knockdown (KnD) cells, no reduction in PI(4)P and PI(4,5)P_2_ pools were observed, which suggests that EFR3s do not directly affect the phosphoinositide pool in the PM since PI4K still can provide sufficient amounts of these phosphoinositides in the PM. However, they observed a decreased plateau phase in Ca^2+^ levels following stimulation of AT1 with angiotensin II, opposite to control cells. It appeared that in the *EFR3* KnD cells, the AT1 receptor is hyperphosphorylated, which the authors consider responsible for its uncoupling from the Gq11 alpha subunit after stimulation. However, it still maintains its ability to be internalized. In their opinion, in *EFR3* KnD cells, a defect in returning the receptors from their desensitized state occurred [18]. The same authors found a similar, although less pronounced, effect on the endogenous adrenergic β-receptors after stimulating *EFR3* KnD HEK cells with isoproterenol, measuring cAMP production. However, there are no detailed data on the molecular mechanism governing the regulation of phosphorylation of GPCRs in which EFR3 proteins are involved.

### 2.3. EFR3A and Insulin-Mediated Dispersal of GLUT4

Vital for glucose homeostasis in animal organisms is insulin-regulated glucose transport into muscle and adipose cells. It depends on GLUT4 (glucose transporter type 4), an integral, 12-transmembrane domain protein responsible for the facilitated diffusion of glucose into cells. Insulin binding to its receptor triggers a phosphorylation cascade involving PI3K, AKT, and mTORC2 that results in several vesicular traffic events leading to the delivery of GLUT4 to the PM (recent review see [24]). Failure to target GLUT4 in the PM results in insulin resistance and type 2 diabetes [25]. In unstimulated adipocytes, most of the GLUT4 pool resides in the intracellular vesicular compartment, including the TGN (trans-Golgi network), endosomal compartment, and insulin-responding vesicles (IRV) [26]. The latter fuse with the PM in a Rab13-dependent manner [27]. Apart from insulin-regulated delivery of GLUT4-bearing vesicles to the PM, insulin plays a role in the regulation of GLUT4 dynamics in the PM. Stenkula et al. [28] suggest that in the PM, GLUT4 occurs as freely diffusing monomers and stable domains containing GLUT4 clusters, which result from fusion with ”selective retention”. According to their model, insulin causes exocytosis of GLUT4-bearing vesicles, leading to monomeric GLUT4 in the PM, which transports glucose into the cell and above-mentioned domains containing GLUT4 clusters. The generation of monomeric GLUT4 outweighs selective retention by 30 times. Retrograde clathrin-mediated endocytosis also takes place from the preexisting clusters. Therefore, the above-mentioned clusters are the hub between functional monomers and the re-internalization of GLUT4.

Ectopically expressed human GLUT4, although distributed to the plasma membrane, did not support the growth of a yeast strain lacking hexose transporters unless this strain had a mutation in the *EFR3* gene (fgy1-1) [29,30,31]. These results pointed the researchers’ attention to the role of EFR3 protein and PI4KA in regulating insulin-stimulated glucose transport and GLUT4 dispersal in 3T3-L1 adipocytes. *EFR3A*, a dominant paralog expressed in these cells (500-fold higher mRNA level when compared to EFR3B) [32], is suggested to function as a key regulator of GLUT4 dispersal in the PM. Knockdown of *EFR3A* or *PI4KA* impairs insulin-stimulated glucose transport in adipocytes. Knockdown of *EFR3*A expression reduces insulin-mediated spreading of GLUT4 in the PM. These data may shed light on the participation of the EFR3A–PI4KA axis in the regulation of GLUT4 function and the role of genes encoding this multiprotein complex in the regulation of GLUT4 function and possibly type 2 diabetes [33].

### 2.4. Brain-Specific EfrA Knockout Promotes Hippocampal Neurogenesis in Mice

Brain-specific deletion of *Efr3a* revealed enhanced hippocampal neurogenesis in adult mice. It appeared that in *Efr3a* KO mice, hippocampus newborn neurons were characterized by extended survival and decreased levels of apoptosis. The probable mechanism is connected with the increased levels of the expression of brain-derived neurotrophic factor (BDNF) and tropomyosin-related kinase B TrkB (TrkB)-encoding genes that govern signaling pathways controlling survival, and first of all, the AKT pathway [34].

Other reports suggest that the EFR3A protein may also play a key role in the irreversible spiral ganglion (SGCs) degeneration in the cochlea. Observation of expression of the mouse *Efr3a* gene during drug (kanamycin and furosemide)-induced hair cell loss and spiral ganglion degeneration indicated a temporal increase in *Efr3a* expression [35]. Further studies using *Efr3a* knockdown mice and mice overexpressing this gene led to the conclusion that loss of *Efr3a* expression may lead to a delay in hair cell loss and spiral ganglion degeneration. The molecular mechanism underlying this effect relates to the abovementioned increased expression of BDNF signaling pathway genes [36,37].

## 3. Association of EFR3A Protein with Disease States

Data concerning the involvement of the *EFR3A* gene and/or protein in human pathology are scarce. However, the literature indicates the involvement of this gene in several types of human disorders, i.e., neurological, cardiovascular, and several tumors.

Neurological disorders include autism spectrum disorders (ASD), in which six somatic, nonsynonymous mutations in the *EFR3A* coding sequence were observed twice more frequently in patients than in control subjects, as was found in a large cohort exome and Sanger sequencing-based study. The fact that the *EFR3A* expression pattern is shared with ASD-associated genes i.e., synaptic genes and PI(4,5)P_2_ phosphatase substantiates the correlation between EFR3A and ASD [38]. *EFR3A* was found among the fourteen genes as the features of primary glioma, allowing the stratification of these patients into low- and high-risk groups [39]. EFR3A was also found to be engaged in essential tremor (ET), a common neurological disorder. Analysis of publicly available and the authors’ own RNAseq data indicated *EFR3A* among seven genes whose expression pattern was abnormally changed, i.e., EFR3A was upregulated [21]. Other reports suggest that the EFR3 protein may also play a key role in spiral ganglion cell (SGCs) degeneration in the cochlea [36,37]. Irreversible sensorineural hearing loss is caused by irreversible damage to cochlear hair cells and subsequent progressive degeneration of spiral ganglion neurons (SGNs).

Interestingly, *EFR3A* is associated with the development of Alzheimer’s disease. The knockout of *Efr3a* in the mice CA3 area of the hippocampus led to Aβ (Amyloid β)-induced depletion of PI(4,5)P2. Moreover, selectively deleting Efr3a at the presynaptic site in CA1 pyramidal neurons leads to improved cognitive function and memory in the APP/PS1 mice model of Alzheimer’s disease [40]. On the other hand, the depletion of highly enriched isoform Efr3b in the CA2/CA3 hippocampal area of pyramidal neurons (PN) results in excitability and social novelty recognition deficits in mice [41].

There are also reports linking EFR3A with cardiovascular diseases, in particular with coronary artery disease (CAD), as *EFR3A* was found to be one of three genes regulated by miR-367. *EFR3A* and *CD69* were significantly upregulated while *FBXW7* was downregulated in the sera of these patients. *CD69* encodes the transmembrane Ca^2+^-dependent lectin-type receptor, and *FBXW7* encodes the F-box protein (containing the F-box, an approximately 40 amino acid residue motif), which constitutes one of the four subunits of SCFs (SKP1-cullin-F-box), the ubiquitin ligase complex. Further experiments on human aortic endothelial cells led to the conclusion that EFR3A and FBXW7 may participate in the regulation of NF-κB-activated inflammatory pathways, possibly via modulation of mTOR/AKT pathways [42].

A whole exome and targeted sequencing study in patients with acute coronary syndromes (ACS) subjected to antiplatelet therapy (after artery opening) revealed that a single nucleotide polymorphism (SNP) in the *EFR3A* loci (rs4736529) was among SNPs in eight genes showing strong associations with 18-month major adverse cardiovascular events (MACE) [43].

Data on the importance of *EFR3A* and its product in neoplastic diseases concern colorectal, pancreatic ductal, nasopharyngeal carcinomas, and brain tumors. In the adenoma case, a novel missense variant in the *EFR3A* gene (chr8: 133057465; p.G390E) was found. However, this was not validated in the cohort of 288 colorectal cancer (CRC) cases. It was not determined whether this variant was a rare nonsignificant mutation in adenoma or was a low-frequency change overlooked by Sanger sequencing [44].

Analysis of the genetic alterations in *EFR3A* in human neoplasms (cBioportal cancer genomics datasets) revealed that pancreatic ductal adenocarcinoma (PDAC) exhibits the highest *EFR3A* amplification frequency (almost 12% of cases) compared to, e.g., nonsmall cell lung cancer (NSCLC) and colorectal cancer (~5%). Patients with *EFR3A* gene alterations (mainly amplifications) presented lower overall survival (~26%) and reduced by ~53% disease-free period compared to subjects with near-normal *EFR3A* expression levels [45]. The increased expression level of *EFR3A* was observed mainly in subjects with mutations in the *KRAS* gene, which is observed in 85% of PDAC patients [46,47]. When EFR3A gene HEK-HT cells harboring KRAS^G12V^ were disrupted, a drastic decrease in PI(4)P, phosphatidylserine, and KRAS levels at the PM was observed, which resulted in reduced RAS signaling. The effect was even stronger after both *EFR3A* and *EFR3B* genes were targeted with sgRNA [47]. The authors suggested the PI4KA pathway as a possible drug target in KRAS mutation-positive PDAC cases and proposed using, e.g., simeprevir, a known antiviral drug, or C7, PI4KA inhibitors [45,47].

circEFR3A (hsa_circ_0135761) was detected among the top dysregulated circRNAs in nasopharyngeal carcinoma (NPC) cases [48]. Further studies confirmed the overexpression of circEFR3A in human NPC cell lines and indicated that circEFR3A promotes the expression of *EFR3A*. The silencing of circEFR3A had an anti-oncogenic effect, which was manifested in increased apoptosis and decreased cell proliferation [49]. The putative role of the oncogenic circEFR3A in the *EFR3A* expression regulation may become a drug target for NPC. However, the limitations concerning circRNA research are still valid [50]. As mentioned above, EFR3A may also be involved in brain tumors. The study which utilized the data from the CRISPR-Cas9 lethality screens [51] performed on patient-derived glioma stem cells indicated a dependency on EFR3A at least in a single mesenchymal GSC-0131 line. Moreover, further analysis of these cell lines suggested a strong genetic redundancy between *EFR3A* and *EFR3B* in these tumors [6].

## 4. EFR3 Structural Features: Atomic Structure and Domain Organization

**Figure 2:**
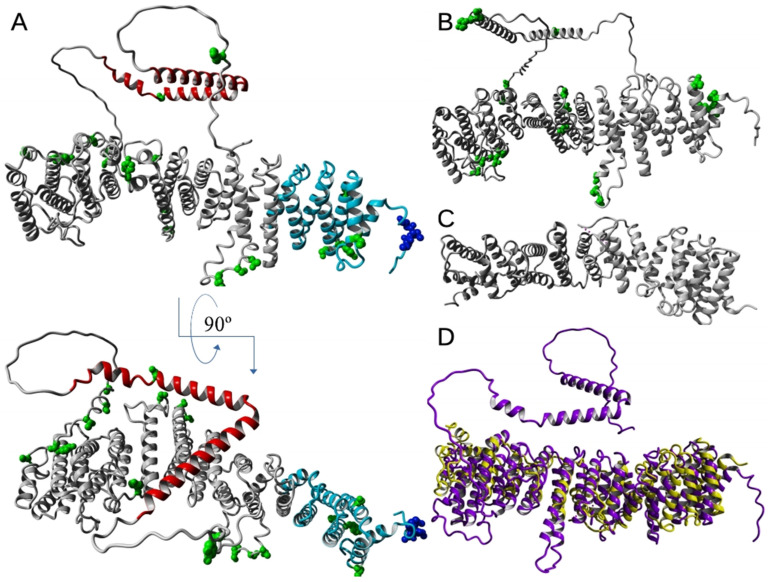
Structural models of EFR3 proteins. Computed structure model of (**A**) human EFR3A (AlphaFold DB: AF-Q14156-F1), cysteine residues marked with blue, phosphorylation sites in green, fragment comprising first 150 amino acid residues at N-terminus marked with cyan, C-terminal part of the protein in red and (**B**) EFR3B (AlphaFold DB: AF-Q9Y2G0-F1), phosphorylation sites in green [60]. Experimental structure (**C**) of Saccharomyces cerevisiae EFR3 (PDB ID: 4N5A) [52]. Overlay (**D**) of structures presented in A (magenta) and C (yellow). Structures rendered with YASARA [61].

Examples of detailed models and experimental atomic structures of EFR3 proteins available in the Protein Data Bank are shown in Figure 2A–C. The crystal structure of the EFR3 protein was first proposed by Wu et al. in 2014 that was performed on the yeast, *Saccharomyces cerevisiae* protein, more precisely on the N-terminal fragment of the EFR3 protein (EFR3-N8–562) [52] except for the disordered loop containing residues 217–232 at 3.2 Å resolution (Figure 2C, PDB ID: 4N5A) by using SAD (single-wavelength anomalous dispersion method) with selenomethionine-substituted crystals for phasing. From their studies, it is known that the N-terminal fragment EFR3-N8-562 is predominantly α-helical, in which the first ~150 residues are folded in 8 helices (H1–H8), being similar ARM or HEAT motifs, and forming a superhelical VHS (Vps-27, Hrs and STAM) domain [53,54,55,56,57]. Residues 48–144 form an ARM (armadillo) motif [58], which consists of a right-handed superhelix formed of several α-helices, a characteristic feature of the EFR3 protein family. In contrast, the remaining part of the molecule comprises 19 alpha-helices being HEAT motifs. The entire molecule of EFR3-N8–562 forms an extended 120 nm rod stabilized via hydrophobic interactions of the helices of one repeat making contact with their counterparts in the neighboring repeats, which results in forming a continuous α-α-superhelix [59]. A conserved, basic patch at an N-terminal part of the molecule (VHS domain) was proven to be responsible for interactions with phosphatidyl inositols [52]. The authors suggest that these interactions are responsible for the attachment of EFR3 and its complexes with other proteins to the PM. Another basic patch occurs near the middle part of the studied molecule, by the loops joining helices 11–18. The abovementioned studies allowed the application of a sophisticated Alpha-Fold software to propose structural models of vertebrate EFR3 molecules (for example, the human EFR3A structural model is shown in Figure 2A (AlphaFold DB: AF-Q14156-F1) and EFR3B structure is shown in Figure 2B (AlphaFold DB: AF-Q9Y2G0-F1). As can be seen, the obtained models are remarkably similar to the original template molecule (see Figure 2D). This indicates the remarkable level of conservation of the secondary and tertiary structures, while only the N-terminal region (residues 11–109 EFR3 of yeast and 14–111 of human EFR3A) molecule shows the substantial degree of amino acid residue sequence identity (23%) and similarity (58%).

**Figure 3:**
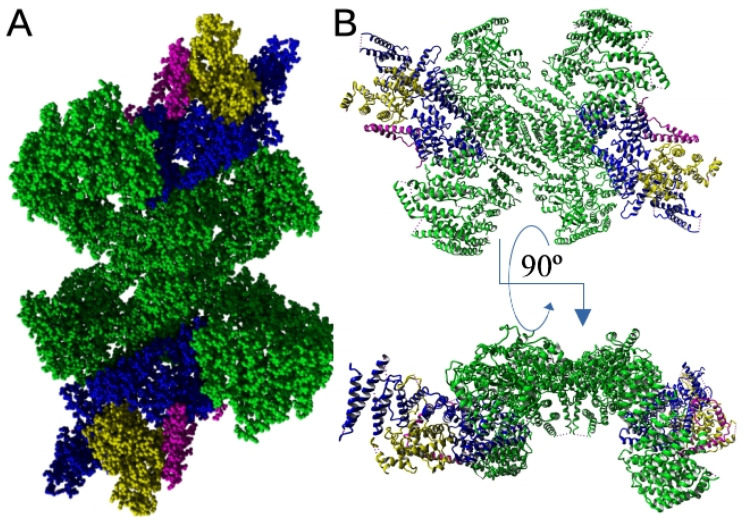
Full atom (**A**) and secondary structure (**B**) representation of PI4KA complex bound to C-terminus of EFR3A as determined via electron microscopy with [64] 3.65 Å resolution (PDB ID: 9BAX). Green, two molecules of PI4Kalpha that form central tandem; blue, TTC7B; yellow, FAM126; magenta, C-terminus of EFR3A. Structures rendered with YASARA [61].

From the first structural studies, it was proposed that the C-terminal part of the EFR3 molecule and its metazoan orthologs form mostly random coil structures [62,63]. However, cryo-EM, HDX-MS, and recent mutational studies of Suresh et al. [64] showed that the conserved middle C-terminal region of the EFR3A molecule (residues 724–787) forms a V-shaped structure composed of three α-helices which allows interaction with its partner proteins that allows PI4K interaction with the PM (see below Section 6.1 and Figure 3).

## 5. Post-Translational Modifications

### 5.1. S-Palmitoylation

S-palmitoylation is a well-known post-translational modification catalyzed mainly by enzymes representing a zinc-finger DHHC motif-containing protein family that is supposed to be responsible for numerous proteins’ interaction with a membrane bilayer and, therefore, subcellular localization of proteins in eukaryotic cells. S-palmitoylation could also be responsible for the stabilization of protein conformation, permitting protein–protein interactions. It is also thought to play a role in several crucial signaling pathways [65,66]. The palmitoylation process of some proteins was found to be engaged in human pathologies, therefore, palmitoylation inhibitors are considered potential therapeutics [67].

Vertebrate EFR3A is known to be palmitoylated. In the N-terminal region, a cluster of 3–4 cysteine residues that are strong candidates for palmitoylation is easily detected with several in silico predicting tools, e.g., CSS-PALM [68], (see Figure 1C and Figure 2A) or found in the proteomic palmitoylation database [69]. EFR3B also has a triple cysteine motif (C5, C7, and C8), a target of palmitoylation. An identical/similar motif is present in *C. elegans* and *D. melanogaster* orthologs of EFR3. Both proteins were found to be metabolically radiolabeled with [^3^H]palmitate [18]. The arrangement of palmitoylated groups in EFR3B affects the interactions between EFR3B and the transmembrane protein, TMEM150A [70]. Interestingly, the same authors found that this post-translational modification, in contrast to the other palmitoylated proteins is stable, i.e., in the case of EFR3B, it was stable over several hours. Similarly, our attempt to pharmacologically inhibit EFR3A palmitoylation with 2-bromo-palmitate (2-BrP) in HeLa cells, even for 48 h treatment was unsuccessful (Trybus et al.—unpublished data). Data from mutational experiments indicate that the PM localization of both EFR3A and EFR3B depends on the presence of a palmitoylation motif at the N-terminal region of the proteins [18,71]. The latter authors also reported that a short 37 N-terminal amino acid residue fragment of EFR3A can target GFP protein to the membrane. This construct is retained in the Golgi network, suggesting that other regions of this protein are responsible for further transport to the PM [18]. On the other hand, data on yeast EFR3 protein suggest stable PM localization despite the absence of an N-terminal ~140 amino acid residue segment containing 3–4 cysteine palmitoylation motif. We have to keep in mind that there are isoforms of EFR3A and EFR3B (isoforms 2) that do not have this cysteine motif (see Figure 1B). So far, we are unaware of existing experimental data on the localization of these isoforms in the PM.

### 5.2. Phosphorylation

According to the data reported in the *PhosphoSitePlus* database, 19 phosphorylation sites can be found in the EFR3A, including three tyrosine residues. At least six S/T residues are present within the short region between S680 and S738, including S694, detected as phosphorylated in numerous reports [72] and S738 in the helical region interacting with TTC7B and FAM126A (see above and [64]). In yeast, phosphorylation was found to inhibit the binding of Ypp-1 (ortholog of TTC7) and, as a consequence, the recruitment of yeast PI4K homolog, Stt4, to the PM [52]. It should be mentioned that although EFR3 orthologs and paralogs differ in the exact phosphorylation sites, one can see some similarities. Namely, certain regions of both molecules contain residues that are the target of phosphorylation, i.e., residues 219–224 in EFR3A and residues 212–216 in EFR3B and in the C-terminus residues 680–738 in paralog A and 689–723 in paralog B, while the other phosphorylation sites are not conserved [72] (see also Figure 2). In yeast EFR3, phosphorylation sites are located mostly within the C-terminal region, i.e., 23 of 25 S/T/Y residues are located downstream of position 580. The phospho-cluster seems much wider than in vertebrates as 19 S/T/Y residues are located between residues 611 and 771 (Figure 2C).

### 5.3. Other Post-Translational Modifications

Both EFR3A and EFR3B are subject to post-translational modifications such as ubiquitination at K residues. In the *PhosphoSitePlus* database [72], the number of sites for this modification varies, from 9 out of 11 K residues in EFR3A to 3 out of 8 K residues in EFR3B. There are no available experimental data concerning the particular function of this modification. Also, there are no experimental data on the function of acetylation of three out of eight K residues that were found to be a target of acetylation in EFR3B and a single K residue detected in EFR3A [72].

## 6. Functional Complexes

**Figure 4:**
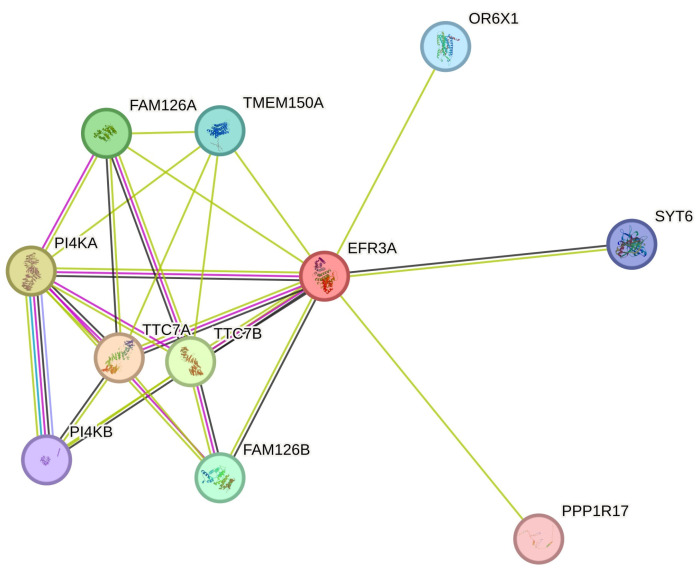
EFR3A known interactions—simplified interactome from String database [73]. Line color code as in the String database, i.e., blue-green, data from the curated database; magenta, experimentally determined; green, red, and dark blue, gene neighborhood, gene fusions, gene co-occurrence, respectively light green, text mining; black, co-expression; and blue, protein homology. For other details, see the String database.

The interaction network of human EFR3 proteins has not been explored in detail. The STRING database shows only several experimentally confirmed partner proteins (Figure 4) for EFR3A. There are indications of numerous other interactions, but they still await further confirmation [73]. Interesting results were obtained using BioID proximity assay technology (review [74]). Using this technique on the osteosarcoma cell line U2OS to study the proteome of focal adhesions, Dong et al. found EFR3A as a new partner for kindlin-2, a stress fiber and focal adhesion integrin regulating protein [75]. Using a similar approach, Counter’s team discovered EFR3A as a KRAS oncogenic (G12V) variant protein partner. Their further experiments documented the in vitro interaction of these proteins and that KO of *EFR3A* and *EFR3B* or *PI4KA* reduced cell proliferation signaling and colony formation of cultured cancer cells accompanied by reduced oncogenic KRAS localization at the PM due to the reduced content of PI(4)P and phosphatidylserine (PS). Moreover, they showed the reduction of KRAS-dependent cancer cell proliferation via PI4KA inhibition as Compound 7 (C7, PMID: 24366037) or Simeprevir, an anti-Hepatitis C virus (known as PI4KA inhibitors) mislocalized KRAS from the PM, indicating the potentiality of these compounds to be repurposed towards anti-cancer drugs [47].

### 6.1. PI4K Anchoring in the Plasma Membrane

The best known and the most important function of the yeast Efr3 protein (or EFR3A/B in humans) is the PM anchorage of phosphatidylinositol-4 kinase (PI4KA in humans or Stt4 in yeast), the key enzyme of the PM phosphoinositide metabolism [76,77,78]. It was found that EFR3A, together with other proteins (see below), forms a PI4KA/Stt4 membrane-anchoring complex [18]. Thus, one of the best-known roles of EFR3 proteins is participation in the metabolism of phosphatidylinositol phosphates, in particular phosphatidylinositol 4-phosphate (PI(4)P), which is one of the most important membrane regulatory phospholipids.

Phosphatidyl inositol phosphates play a fundamental role in cellular signaling, being involved in the regulation of growth and proliferation, migration, and survival (for recent review, see e.g., [79]). Examples of known signaling pathways are numerous, starting from phosphatidyl inositol (3,4,5)-trisphosphate (PI(3,4,5)P_3_) and phosphatidyl inositol (4,5)-bisphosphate (PI(4,5P)_2_) that are the sources of diacylglycerol DAG and inositol trisphosphate IP3 to PI(3,4)P_2_ engaged in growth factor, mTOR, and AKT signaling to various PIP derivatives engaged in endocytosis. Phosphatidyl inositol phosphates are most abundant in the PM but are also present in the Golgi complex, the endosomal, and the lysosomal system [80,81]. Both yeast and higher eukaryotic PI4Kinases are large proteins with a subunit molecular weight of 214 kDa (1900 amino acid residues in yeast) [82] and 240 kDa (2102 amino acid residues in humans) [83]. This enzyme was found to be essential for yeast cell survival, but its deletion was rescuable at 1 M sorbitol, according to its discoverer group [84]. However, other researchers working with different strain backgrounds found this enzyme essential under all conditions [85]. On the other hand, in *C. albicans*, PI(4)P and, thus, PI4Kinase were found not necessary for survival but appeared indispensable for cell wall synthesis and infectivity [86].

The tetratricopeptide repeat domain 7 (TTC7A/B, [87,88]) are 858 and 848 amino acid residue proteins encoded by two paralog genes, located in chromosomes 2 and 14, respectively. Tetratricopeptide (TPR) domains are 34 amino acid residue repeating units forming two antiparallel α-helices separated by a turn that forms a structure resembling a spiraling staircase [87,88]. TTC7 (similar to yeast equivalent, Ypp1) is responsible for maintaining the integrity of the complex and is thought to be responsible for the correct folding of the complex [62,89]. Mutations in *TTC7A* have been associated with the rare hereditary human disease, combined immunodeficiency with multiple intestinal atresias (CID-MIA) [90,91,92,93,94]. Mutations in *TTC7B* have also been inferred in the molecular basis of several disorders, e.g., hypomyelinating leukoencephalopathy [95].

Proteomic analysis of TCC7 protein partners showed that FAM126A/B, also known as hyccin, or HYCC1 (hyccin PI4KA lipid kinase complex subunit 1) belongs to the complex [62]. FAM 126A, a 521 amino acid residue protein, is present only in higher eukaryotes, and there is no ortholog protein/gene in *S. cerevisiae*. Mutations in the gene encoding this protein resulting in the absence or presence of dysfunctional HYCC1 were reported to be connected with hypomyelination and congenital cataracts. Patients lacking the FAM126A protein and with L53P and C57R substitutions showed a reduction in the levels of PI4KA and its adapter proteins TTC7A/B and EFR3A. This suggests the disorganization of the complex in the absence of FAM126A. A decrease in PI(4)P was also observed in fibroblasts from hypomyelination and congenital cataract patients [62,96,97]. It should be noted that the homologous gene, *FAM126B/HYCC2,* encodes the other member of the PI4 kinase complex paralog protein, but there are no experimental details apart from the expression profile. However, a recent study on colorectal cancer indicates the loss of expression of *FAM126A* in 7% of cases due to promoter hypermethylation. These cancer cells become dependent on *FAM126B* expression [98].

As was mentioned, PI4KA/Stt4 in eukaryotic cells is anchored to the PM by the evolutionarily conserved giant protein complex containing TTC7A/B (or Ypp1), FAM126A [62], and EFR3A/B (EFR3 in yeast) [89]. Data from cryo-EM analysis of the structure (at 3.6 Å resolution) showed that 700 kDa super-assembly is formed of heterotrimers assembled from PI4KA, TTC7, and FAM126 [62,99]. The complex (also called complex I) has a flat surface perfectly adapted to the PM bilayer interface. Catalytic sites of the kinase are directed towards PM-localized PI [63]. It is suggested that interaction between PI4KA and TTC7/FAM126 helps to stabilize the conformation of the PI4KA and to enable the formation of an interaction surface with the membrane, including the kinase active sites. The conserved common surface in TTC7/FAM126 parallel to the PI4KA membrane interaction surface serves as a docking site for the abovementioned C-terminally located, V-shaped domain of EFR3, which is responsible for PM attachment of the complex [63,64,89] (Figure 3).

There is also an alternative way to connect PI4KA to PM via complex II. Namely, this is mediated by TMEM150, a conserved mammalian ortholog of the yeast Sfk1 protein belonging to the TMEM150/FRAG1/DRAM family, which was found to substitute TTC7 without changing the localization of PI4KA or EFR3A in the PM [100]. However, attachment to TMEM150, at least in the case of EFR3B homolog, depends on its palmitoylation pattern. More specifically, the form of EFR3B triple palmitoylated on residues C5, C7, and C8 or double palmitoylated on residues C5 and C7 or C8 forms complex I occurring in the lo region and is responsible for the synthesis of PI(4)P and PI(4,5)P_2_ in these domains. Double palmitoylation of EFR3B at residues C7 and C8 enables its interaction with TMEM150A and the formation of complex II and the associated relocation of TMEM150A from lo to ld and the synthesis of PI(4)P and PI(4,5)P2 in the ld PM regions [70]. A similar mechanism likely governs switching between alternative protein complexes and, therefore, PM domains by EFR3A, but it remains to be discovered.

### 6.2. EFRA Possibly Plays a Role as Membrane Raft Organizer—Interaction with Flotillin-2

PMs of living cells are characterized by several physicochemical parameters, e.g., phase state, mobility of component molecules, and mechanical properties. The phase state behavior of the PM is characterized by lateral heterogeneity as was demonstrated via a variety of biophysical and biochemical methods. The existence of membrane (lipid) rafts is one of the best-known forms of this heterogeneity first proposed by Simons and Ikonen in 1997 [101]. The basic assemblies, called resting-state rafts, are cholesterol-enriched, more ordered domains that are small (~20 nm in diameter), dynamic (Ʈ1/2 ~1 s), and contain a set of characteristic proteins (for reviews, see e.g., [102,103,104]). The specific set of membrane proteins, including membrane proteins belonging to the SPFH family (stomatin/prohibitin/flotillin/HflK), such as raft scaffold proteins flotillin-1 and flotillin-2, stomatin, or stomatin-like protein, seem to be permanent components of membrane rafts [105]. These proteins share a common feature: they associate with raft domains, possibly through cholesterol-binding and/or oligomerization [106,107]. Besides the mentioned proteins, rafts also include other palmitoylated and transmembrane proteins and GPI-anchored proteins (for a review, see [108,109]). In general, raft domain(s) are engaged in many physiological processes underlying various functions of organisms, such as numerous signal transduction pathways [110,111], several innate and adaptive immune responses, and well-known host-pathogen interactions (e.g., [112,113]), neoplasia (e.g., [114,115,116,117]), and other pathologies, in particular neuropathologies such as atherosclerosis and Alzheimer’s disease [118,119].

Results of our work on the lateral membrane organization support the idea that the underlying molecular mechanism of resting state raft organization is, to a large extent, protein–protein interactions [102,120], particularly those involving flotillins and other palmitoylated proteins. We have previously found that MPP1 (membrane palmitoylated protein 1, for review, see [121]) is involved in erythroid cells’ membrane raft organization and regulation in a palmitoylation-dependent manner. Based on experiments on erythroid cell line HEL with *MPP1* KnD, on erythrocyte membranes and recombinant proteins, and using a variety of biochemical and biophysical methods, we discovered that MPP1 may play a key role in resting state membrane raft organization and regulation in erythroid cells. This conclusion was based on three major sets of observations. Firstly, pharmacological inhibition of palmitoylation or KnD of the *MPP1* gene led to the decreased membrane order and changed membrane probe mobility [122,123,124]. Secondly, *MPP1*-knockdown affected the activation of MAP-kinase signaling via raft-dependent tyrosine kinase receptors, particularly from insulin receptors [122,125]. The signal inhibition occurred at the level of H-Ras, as GDP-to-GTP exchange upon insulin treatment was not observed. Moreover, FLIM-FRET microscopy revealed impaired interaction of H-Ras with Raf (effector) in insulin-treated MPP1 KnD cells [118]. Thirdly, MPP1 in the erythrocyte membrane was found to bind the raft-marker proteins, flotillin-1 and flotillin-2 [126]. These proteins are found in high molecular-weight complexes obtained after chemical cross-linking in native erythrocyte membranes, and their presence was shown to be independent of the well-known protein 4.1-spectrin-actin-based complexes of MPP1. These interactions were confirmed via co-immunoprecipitation, pull-down, overlay, and proximity assays. Moreover, our kinetics studies, by using the surface plasmon resonance (SPR) method, indicate high affinity binding to flotillin-1 (K_D_ = 18.1 ± 2.4 nM) and flotillin-2 (K_D_ = 24.8 ± 6 nM). Further studies on recombinant domains of MPP1 binding to flotillins indicate that a short sequence of ~40 amino acid residues between domains D5 and SH3 may be responsible for these interactions [127].

**Figure 5:**
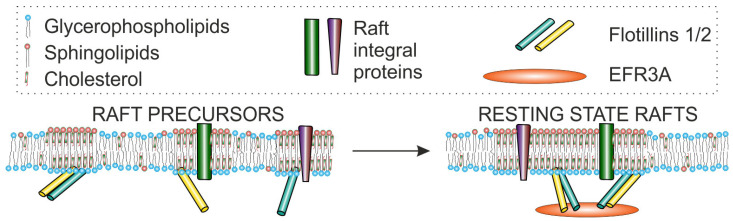
The interaction of EFR3A with flotillins may play a key role in the organization and regulation of resting state rafts. Reprinted from ref. [128].

On the other hand, MPP1 is not expressed at high levels in most of the cell lines of mammalian origin we examined [120]. Our recent studies on HeLa cells were inspired by the idea that other protein(s) may play a role in resting state raft organization and regulation via interaction with raft scaffolding proteins, which are flotillins [128]. We first used a pull-down approach in which flotillin-2 was immobilized on a Sepharose resin and identified DRM (detergent-resistant membrane) fraction-associated proteins via the MS/MS technique. DRMs are considered a biochemical representation of membrane rafts, a kind of “read-out of whether a protein is likely to associate with rafts” [129]. Among several proteins bound to the resin, EFR3A appeared as a strong putative flotillin-2 partner. This result was confirmed via immunodetection of EFR3A and co-immunoprecipitation. The same pull-down results were obtained on several other cells, e.g., LNCaP and PC-3. EFR3A was present in the DRM fraction, and this presence was cholesterol-dependent. Further experiments indicated a substantial decrease in PM order and change in membrane raft probe mobility as revealed via FLIM and svFCS of living HeLa cells with an *EFR3A* KnD. Moreover, changes in membrane fluidity were observed also in the case of giant PM vesicles (GPMVs), which are supposed to be membrane skeleton and cytoskeleton proteins-free [130]. Moreover, a role of raft-associated EFR3A in cell signaling could be expected, as the EGF receptor (EGFR) function is known to depend on raft association [131] and depends on the EFR3A cellular status. Indeed, cells with a decreased level of that protein showed disturbed phosphorylation of EGFR and PLCγ1 (phospholipase Cγ1) upon stimulation with EGF. A similar effect was observed upon cholesterol depletion via treatment of wild-type HeLa cells with MβCD [128]. In light of the above-referred study, we may suggest that another role of EFR3A was uncovered. Namely, EFR3A may induce flotillin(s) and other protein and lipid components’ oligomerization and, as a result, resting state raft formation (Figure 5). However, we should note that further studies should be carried out to build a complete picture in light of the current discovery of flotillin structure in the membrane environment [132]. The interaction of flotillins with EFR3A in vitro should be characterized, and reconstitution studies to confirm the effect of the protein complex on the physical properties of the membrane should provide more evidence to support our model.

## 7. Concluding Remarks and Prospects

This review summarizes the information regarding the still poorly understood EFR3A protein and its encoding gene. Most data relate to the role of this protein as an element of the PI4KA complex responsible for its anchoring to the plasma membrane. However, it is interesting what the role of isoform 2 of both EFR3 paralog proteins is, as they neither have hydrophobic cluster residues nor are palmitoylated. Data from studies using the BioID technique encourage hypothesizing on diverse physiological roles, especially those related to the role of EFR3A interactions with the mutated KRAS protein. It seems that the data from our laboratory regarding the role of this protein in the organization and regulation of membrane rafts open a new field of research. When it comes to the major functions of this protein in the cell, many questions arise, and the main one seems to be whether the formation of the PI4KA docking complex in the plasmalemma is related to the function of the “raft organizer”. However, we need to wait for further research as the experimental data are still slightly incomplete.

## Data Availability

No new data were created or analyzed in this study.

## Abbreviations

The following abbreviations are used in this manuscript:

ACS	acute coronary syndromes
ARM	armadillo
ASD	autism spectrum disorders
AT1R	angiotensin II receptor type 1
BDNF	brain-derived neurotrophic factor
CAD	coronary artery disease
CID-MIA	combined immunodeficiency with multiple intestinal atresias
CRC	colorectal cancer
DAG	diacylglycerol
DRM	detergent-resistant membrane
EFR3A	eighty-five requiring 3A
GPMVs	giant PM vesicles
IRV	insulin-responding vesicles
NPC	nasopharyngeal carcinoma
PDAC	pancreatic ductal adenocarcinoma
PI(3,4,5)P_3_	phosphatidyl inositol (3,4,5)-trisphosphate
PI(4,5P)_2_	phosphatidyl inositol (4,5)-bisphosphate
PI4KA	phosphatidylinositol 4-kinase alpha
PLCγ1	phospholipase Cγ1
PM	plasma membrane
SAD	single-wavelength anomalous dispersion
SNP	single nucleotide polymorphism
TRP	transient receptor potential
TTC7	tetratricopeptide repeat domain 7
